# Improving MDS Risk Assessment: The Role of Monocytopenia and Lymphocytopenia Beyond IPSS-R

**DOI:** 10.3390/medicina61091689

**Published:** 2025-09-17

**Authors:** Marijana Virijevic, Ljubomir Jakovic, Lazar Trajkovic, Mirjana Cvetkovic, Zlatko Pravdic, Mirjana Mitrovic, Nada Suvajdzic-Vukovic, Andrija Bogdanovic

**Affiliations:** 1 Clinic of Hematology, University Clinical Center of Serbia, Dr. Koste Todorovica 2, 11000 Belgrade, Serbia; ljubajak@yahoo.com (L.J.); trajkovic.lazar33@gmail.com (L.T.); mimamima.cvetkovic@gmail.com (M.C.); zlatko.pravdic@gmail.com (Z.P.); mirjanamitrovic777@gmail.com (M.M.); suvajdzic.nada@gmail.com (N.S.-V.); andrija.bogdanovic@gmail.com (A.B.); 2 Medical Faculty, University of Belgrade, Dr. Subotica 8, 11000 Belgrade, Serbia

**Keywords:** monocytopenia, lymphocytopenia, IPSS-R, MDS

## Abstract

*Background and Objectives*: The revised international prognostic scoring system (IPSS-R) remains the most widely used prognostic tool for myelodysplastic syndrome (MDS). There is growing evidence that inflammation and immunological dysregulation are important in the pathogenesis of MDS. Moreover, monocytopenia and lymphocytopenia are correlated with adverse outcomes in patients with MDS. However, standard guideline-driven diagnostic and prognostic models do not evaluate host immunity parameters. This study explored the prognostic relevance of monocytopenia and lymphocytopenia at diagnosis for overall survival (OS) as medical endpoints independent of IPSS-R. *Materials and Methods:* This retrospective study included 217 patients with MDS diagnosed and treated at the University Clinical Center of Serbia between July 2019 and July 2024. MDS was diagnosed based on the 2016 World Health Organization (WHO) criteria. *Results*: Univariate analysis revealed that patients with monocytopenia (absolute monocyte count (AMC) < 0.3 × 10^9^/L) had adverse outcomes compared to individuals with normal AMC (AMC ≥ 0.3 × 10^9^/L) (median OS with/without risk factor 20 months vs. 60 months, respectively, log rank test *p* = 0.0009). Moreover, lymphocytopenia (absolute lymphocyte count (ALC) < 1.2 × 10^9^/L) was shown to have a significant impact on survival (median OS with/without risk factor 17 months vs. 29 months, respectively; log-rank *p* = 0.0182). In further multivariate analysis, IPSS-R, AMC < 0.3 × 10^9^/L, ALC < 1.2 × 10^9^/L, and DMAs/HSCT were identified as independent prognostic factors for OS (Cox multivariate model, *p* < 0.001, *p* = 0.0237, *p* = 0.006, *p* < 0.001, respectively). *Conclusions:* Our findings suggest that ALC and AMC can serve as readily accessible and verifiable prognostic tools in MDS at presentation. Combined with IPSS-R, these markers may provide additional prognostic insights, enabling better risk stratification in MDS patients who could benefit from future immunotherapies.

## 1. Introduction

Myelodysplastic syndromes (MDS) represent a heterogeneous group of clonal myeloid hemopathies, determined by ineffective hematopoiesis, disordered maturation, genetic abnormalities, peripheral blood cytopenias, and a propensity for progression to AML [1,2].

The starting point in the diagnostic algorithm of MDS is cytomorphological evaluation of peripheral blood and bone marrow aspirate, characterized by dysplasia and percentage of blasts, accompanied by well-defined cytogenetic and molecular aberrations [1,3].

To confirm the diagnosis, a bone marrow trephine biopsy is recommended, which contributes to further information regarding abnormal bone marrow topography, cellularity, and fibrosis. Similarly, flow cytometry abnormalities in myeloid precursors also support the diagnosis of MDS [4].

Currently, both the 5th edition of the World Health Organization (WHO) classification and the International Consensus Classification recognize two main MDS classes: MDS with defining genetic abnormalities (del(5q), *SF3B1* and *TP53* mutation) and morphologically defined MDS based on various cytological and histopathological features [1,2].

Accounting for the vast discrepancy in prognosis and risk of disease progression, several risk stratification systems, ranging from the International Prognostic Scoring System (IPSS) to the revised (IPSS-R) and molecular (IPSS-M), have been developed to provide more personalized treatment strategies for patients with MDS at diagnosis [5,6,7].

Although the IPSS-M offers a higher degree of prognostic accuracy by acknowledging the role of specific gene mutations, IPSS-R remains the cornerstone tool for everyday clinical practice because of its simplicity and availability [6,7].

There is growing evidence that immune dysregulation and bone marrow microenvironment abnormalities play important roles in MDS pathogenesis and progression [8,9,10,11]. Integrating novel immunological data into the IPSS-R and IPSS-R could facilitate a more precise discrimination between MDS risk groups. However, standard guideline-driven diagnostic and prognostic models do not evaluate host immune parameters [5,12,13].

Accordingly, peripheral blood absolute monocyte count (AMC) and absolute lymphocyte count (ALC) are readily available parameters and can be considered surrogates of host immunity. In previously published data, abnormal counts of both AMC and ALC were associated with adverse outcomes in some solid tumors, lymphomas, and conditions characterized by inflammation [14,15,16,17]. Moreover, their prognostic impact on disease progression and poor survival has been observed in other hematological diseases, including myeloproliferative neoplasms and MDS [18,19,20,21,22].

Taking all this into account, it opens the possibility of better selection of patients for higher intensity frontline therapy, including allogeneic stem cell transplantation (ASCT). In addition, the advent of novel immunotherapies may contribute to better outcomes in these subgroups [10].

This study aimed to evaluate the distribution of AMC and ALC in different MDS subtypes and risk groups according to WHO 4th and IPSS-R [3,6] and to analyze the prognostic relevance of monocytopenia and lymphocytopenia for overall survival (OS) as a medical endpoint independent of IPSS-R in a uniform single center group of patients with primary MDS at diagnosis.

## 2. Materials and Methods

### 2.1. Patients

This retrospective study included 217 de novo patients with MDS diagnosed and treated at the University Clinical Center of Serbia between July 2019 and July 2024. The diagnosis of MDS was based on the 2016 WHO criteria and recommendations for the diagnosis of primary MDS [3,23].

Patient risk stratification was performed using the IPSS-R score, which determines management, along with age, comorbidities, and overall fitness [6].

All the patients signed a general informed consent form at the time of diagnosis. This study was approved by the Institutional Review Board of the University Clinical Center of Serbia in accordance with the Declaration of Helsinki and Good Clinical Practice (protocol number 39/26, 26 September 2024).

### 2.2. Clinical Parameters

Medical records were retrospectively reviewed for the following clinical and laboratory data: MDS subtype, age, sex, white blood cell count (WBC), hemoglobin level, platelet count, absolute neutrophil count (ANC), ALC, AMC, peripheral blood (PB) and bone marrow (BM) blast percentage, cytogenetic risk group (according to IPSS-R), transfusion dependence, MDS progression to a higher IPSS-R grade or acute leukemia, and survival outcome.

### 2.3. Assessment of Blood Counts and Absolute Lymphocyte/Monocyte Counts

As part of the routine diagnostic workup all patients underwent complete blood count (CBC) analysis before treatment. Blood counts were measured using a Pentra DX-Nexus analyzer (Horiba Medical^®^, Montpellier, France), following the manufacturer’s standard protocols. Additionally, manual leukocyte differential analysis was conducted on May–Grünwald–Giemsa (Merck^®^, Darmstadt, Germany) stained peripheral blood smears [24]. Clinical laboratory specialists examined 200 leukocytes per sample [25].

Patients with an AMC ≥ 1.0 × 10^9^/L and ≥10% monocytes in PB or a white blood cell count >13.0 × 10^9^/L were excluded, as they were likely to suffer from Chronic Myelomonocytic Leukemia (CMML) or myelodysplastic/myeloproliferative neoplasms [3,26].

A cut-off value of 0.3 × 10^9^/L corresponding to the median AMC reported for healthy Caucasians was used for stratification of patients into monocytopenic (AMC < 0.3 × 10^9^/L) and non-monocytopenic (AMC ≥ 0.3 × 10^9^/L) groups [27].

Patients were stratified into lymphopenic and non-lymphopenic groups, based on the cut-off value of ALC < 1.2 × 10^9^/L, according to proven prognostic relevance in earlier studies [19,20].

### 2.4. Treatment

In total, 60 patients were treated with disease-modifying agents, including 14 who underwent allogeneic hematopoietic stem cell transplantation (allo-HSCT), while the rest received lenalidomide and azacytidine (8 and 38 patients, respectively). Erythropoietin and danazol were used sporadically because of reimbursement/regulatory concerns (three and nine patients, respectively). The remaining patients received the best supportive care.

### 2.5. Statistical Methods

The primary endpoint of the study, OS, was measured from the beginning of treatment until the last follow-up (censored patients were still alive at the time of analysis) or the time of death from any cause. Survival data for transplant patients were censored at the time of HSCT.

Statistical methods of Descriptive statistics (mean, median, and range), Chi-square and Fisher tests, and other nonparametric tests [Mann–Whitney U (MWU) test and Kruskal–Wallis (KW) test] were used. Survival analysis was performed using the Kaplan–Meier method. The statistical significance of the OS differences within groups was estimated using the log-rank test. Multivariate analysis was performed using the Cox proportional hazards model. Differences were considered statistically significant at *p* < 0.05. Tests were performed using TIBCO Statistica statistical software, v13.3 (Palo Alto, CA, USA).

## 3. Results

### 3.1. Baseline and Disease-Related Characteristics

The baseline characteristics (age, gender, peripheral blood values, blast counts in bone marrow and peripheral blood) of 217 patients with primary MDS are shown in Table 1. MDS subtypes are showcased in detail in Table 2. Risk stratification by IPSS-R was very low (22; 10.1%), low (60; 27.7%), intermediate (69; 31.8%), high (44; 20.3%), and very high (22; 10.1%). Adverse karyotype 41/217 (18.9%) were classified as intermediate, poor, or very poor in 24 (11%), five (2.3%), and 12 (5.6%) patients, respectively (Table 2).

Transfusion dependence was documented in 54/217 (24.9%) patients (Table 2). After a median follow-up of 21 months, 96/217 (42.2%) deaths and 40/217 (18.4%) cases of disease progression (leukemic transformation and transformation to high-risk MDS) were documented (Table 2).

### 3.2. Comparison of Patients Stratified by the AMC and ALC

The number of patients with ALC < 1.2 × 10^9^/L was 77/217 (35.5%). A total of 133/217 (61.3%) patients had AMC < 0.3 × 10^9^/L (Table 1).

In comparison to patients with normal AMC (AMC ≥ 0.3 × 10^9^/L), those with monocytopenia (AMC < 0.3 × 10^9^/L) were significantly related to several adverse disease features: leukocyte count (*p* = 0.0001), lower leukocyte count (WBC < 4.0 × 10^9^/L, *p* = 0.0001), lower neutrophil count (*p* = 0.0001), lower ALC (*p* = 0.0001), higher percentage of BM blasts (*p* = 0.005), higher risk IPSS-R (*p* = 0.007), adverse cytogenetic risk groups (*p* = 0.006), transfusion dependence (*p* = 0.049) and inferior survival (*p* = 0.0001) (Table 1 and Table 2).

Lymphocytopenic patients (ALC < 1.2 × 10^9^/L) compared to cases with normal ALC values (ALC ≥ 1.2 × 10^9^/L) were associated with the following disease characteristics: older age (*p* = 0.003), overall leukocyte count (*p* = 0.0001), lower leukocyte count (WBC < 4.0 × 10^9^/L, *p* = 0.001) and disease progression and leukemic transformation (*p* = 0.016) (Table 1 and Table 2).

### 3.3. The Prognostic Relevance of Monocytopenia and Lymphocytopenia for Overall Survival

#### 3.3.1. Univariate Analysis

Univariate analysis included the IPSS-R, two additional host immunity parameters, AMC and ALC, which are not part of the current prognostic stratification models, as well as disease-modifying treatments and transplant.

**Figure 1:**
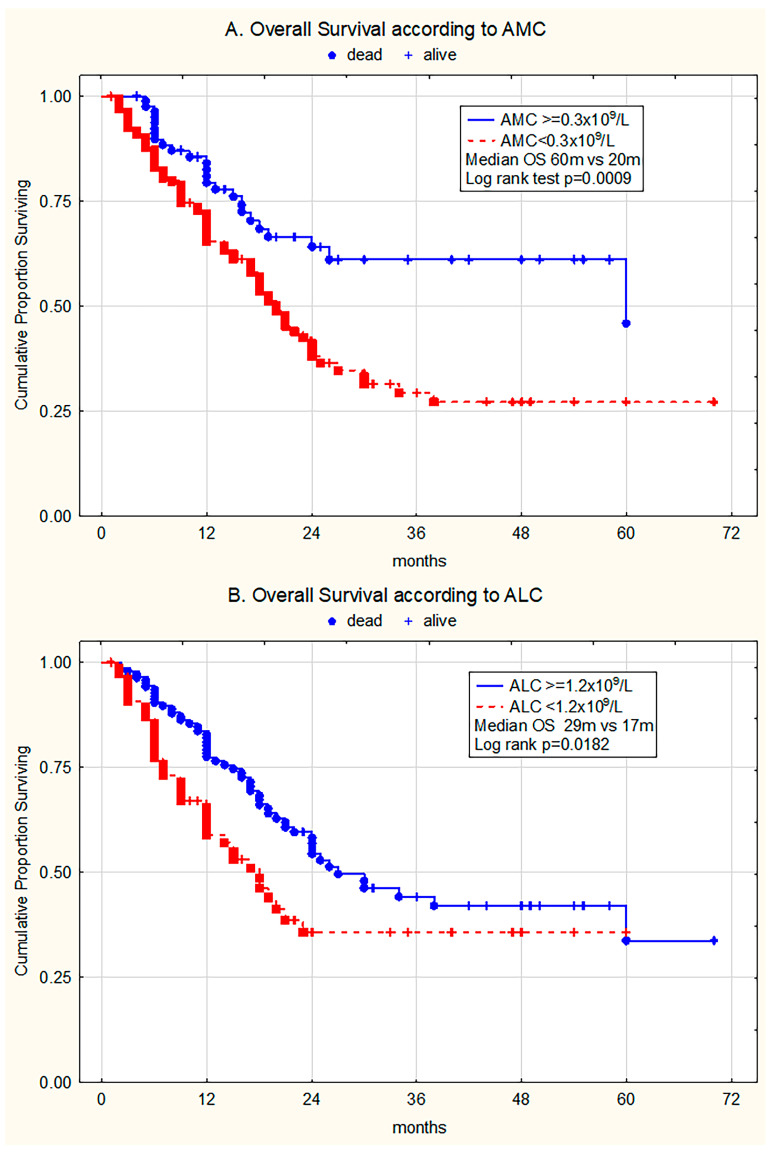
Kaplan–Meier curves estimating (**A**) overall survival (OS) of the whole patient group without and with monocytopenia (AMC ≥ 0.3 × 10^9^/L vs. AMC < 0.3 × 10^9^/L), (**B**) OS of patients without and with lymphocytopenia (ALC ≥ 1.2 × 10^9^/L vs. ALC < 1.2 × 10^9^/L).

Univariate analysis in the whole patient group (n = 217) revealed that patients with monocytopenia (AMC < 0.3 × 10^9^/L) had adverse outcomes compared to individuals with normal AMC (AMC ≥ 0.3 × 10^9^/L) (median OS with/without risk factor 20 months vs. 60 months, respectively, log rank test *p* = 0.0009) (Figure 1A). Moreover, lymphocytopenia (ALC < 1.2 × 10^9^/L) had a significant impact on survival (median OS with/without risk factor 17 months vs. 29 months, respectively, log-rank *p* = 0.0182) (Figure 1B).

**Figure 2:**
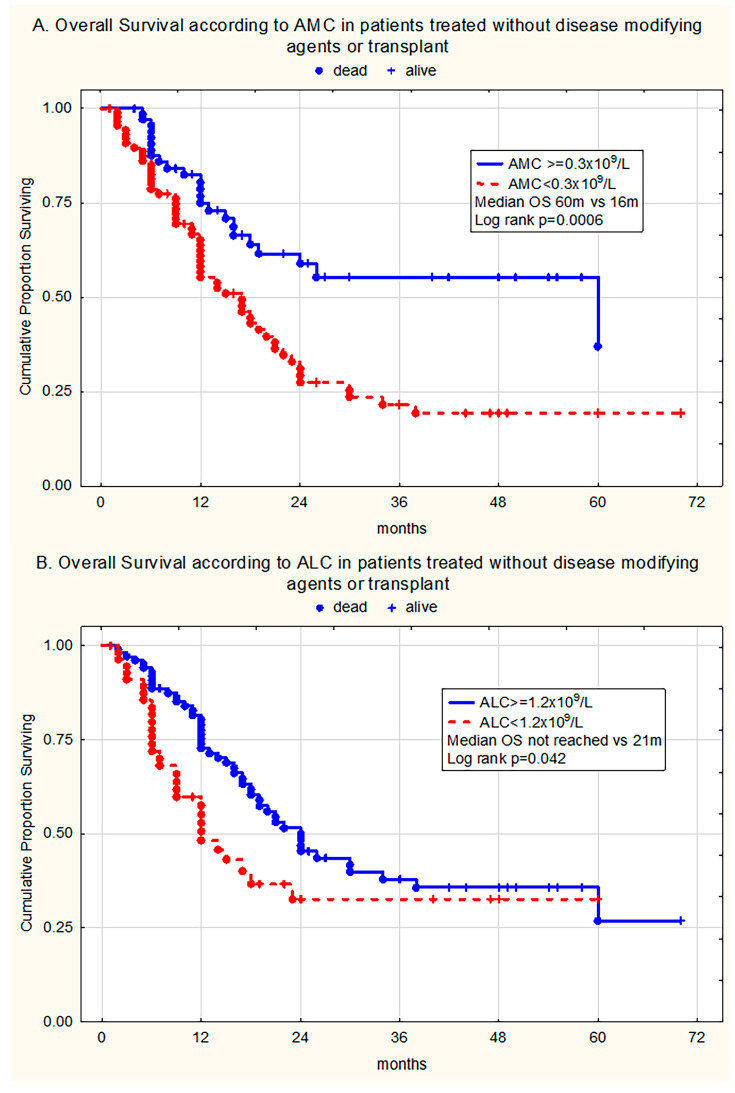
Kaplan–Meier curves estimating (**A**) overall survival (OS) in patients treated without disease-modifying agents or transplantation without and with monocytopenia (AMC ≥ 0.3 × 10^9^/L vs. AMC < 0.3 × 10^9^/L), (**B**) OS of patients without and with lymphocytopenia (ALC ≥ 1.2 × 10^9^/L vs. ALC < 1.2 × 10^9^/L).

Additionally, univariate analysis in the selected patient group which did not receive disease-modifying agents (DMA) or HSCT (n = 157) revealed that patients with monocytopenia (AMC < 0.3 × 10^9^/L) had adverse outcomes compared to individuals with normal AMC (AMC ≥ 0.3 × 10^9^/L) (median OS with/without risk factor 16 months vs. 60 months, respectively, log rank test *p* = 0.0006) (Figure 2A). Moreover, lymphocytopenia (ALC < 1.2 × 10^9^/L) had a significant impact on survival (median OS with/without risk factor 21 months vs. not reached, respectively, log-rank *p* = 0.042) (Figure 2B).

The introduction of DMAs/HSCT had a significant impact on survival (median OS with/without therapy not reached vs. 19 months, respectively, log rank test *p* = 0.0006).

**Figure 3:**
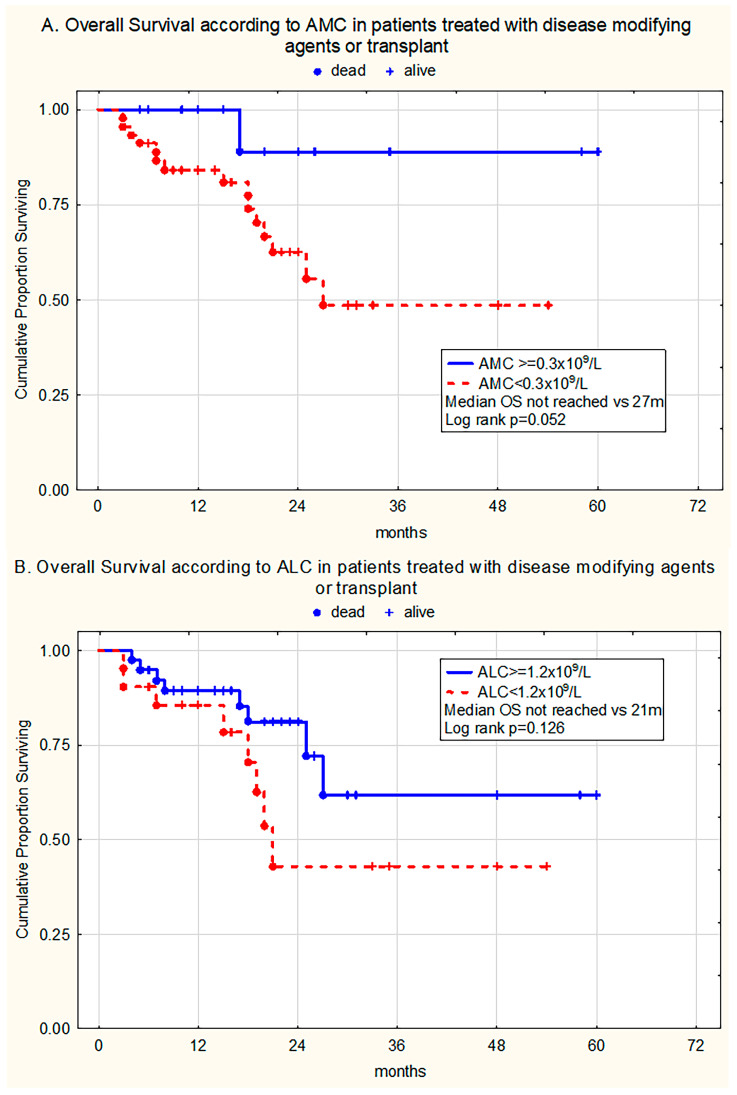
Kaplan–Meier curves estimating (**A**) overall survival (OS) in patients treated with disease-modifying agents or transplantation without and with monocytopenia (AMC ≥ 0.3 × 10^9^/L vs. AMC < 0.3 × 10^9^/L); (**B**) OS of patients without and with lymphocytopenia (ALC ≥ 1.2 × 10^9^/L vs. ALC < 1.2 × 10^9^/L).

In the group treated with DMAs/HSCT (n = 60), monocytopenia retained borderline statistical significance (*p* = 0.052), while lymphocytopenia did not influence survival (*p* = 0.126) (Figure 3A,B).

#### 3.3.2. Multivariate Analysis

In further multivariate analysis, IPSS-R, AMC < 0.3 × 10^9^/L, ALC < 1.2 × 10^9^/L, and DMAs/HSCT were identified as independent prognostic factors for OS (Cox multivariate model, *p* < 0.001, *p* = 0.0237, *p* = 0.006, *p* < 0.001, respectively) (Table 3A). In a second multivariate model including IPSS-R > 3, AMC < 0.3 × 10^9^/L and ALC < 1.2 × 10^9^/L in patients not treated with DMAs/HSCT, all factors retained statistical significance as independent prognostic factors (Cox multivariate model, *p* < 0.001, *p* = 0.0329, *p* = 0.0184, respectively) (Table 3B).

## 4. Discussion

Recent publications indicate that non-malignant immune cells and their dysregulation are not only a consequence of MDS but also have an active effect on biological diversity and could contribute to disease progression and clinical outcomes [28]. In light of this, novel evidence suggests a key role for innate immune- and inflammation-related pathways in MDS pathogenesis [9].

Macrophage dysfunction in the BM microenvironment is correlated with the development of MDS. The main mechanisms include impaired phagocytosis and induction of pro-inflammatory pathways, leading to disrupted normal hematopoiesis and accelerated disease severity [29,30]. Conversely, malignant clonal expansion has been associated with T-cell phenotype population abnormalities, which result in reduced immune surveillance, reduced cytotoxic potential of T cells, and disease-mediated immunosuppression [31,32].

The practical usefulness of immune dysregulation in MDS has been analyzed in several clinical studies. These studies reported inconsistent results regarding the effects of monocytopenia on patient outcomes.

For instance, Saeed et al., in their study including 889 patients with MDS, demonstrated a negative impact of monocytopenia and lymphocytopenia (<0.3 × 10^9^/L, <1.2 × 10^9^/L, respectively) on survival outcomes in univariate analysis. However, their findings were not confirmed by further multivariate statistics, showing borderline significance when the IPSS-R was introduced [20]. Further support for these results was presented in the investigation of the Greek MDS registry on 1719 patients, where inferior survival correlated with monocytopenia (<0.2 × 10^9^/L) in both uni- and multivariate models [22].

These findings are consistent with the results of the present study, highlighting the role of monocytopenia as an adverse risk factor for OS in patients with MDS.

In contrast, several reports have implied that monocytosis is associated with unfavorable outcomes in patients with MDS. These studies are largely based on analyses from the Düsseldorf MDS registry, which includes patient data dating back to 1982 [21,33]. They noted that monocytosis (reported as AMC > 0.6 × 10^9^/L and AMC > 0.4 × 10^9^/L) was significantly associated with shorter OS. However, the authors highlighted that the majority of the analyzed MDS cases with a higher monocyte count would now be classified as CMML, according to both the 5th WHO classification and ICC [1,2,21]. Simultaneously, in the same report, Silzle et al. also showed a negative effect of monocytopenia (<0.2 × 10^9^/L) on OS [21]. Additionally, Qu et al., in their research on 334 newly diagnosed primary untreated MDS patients, indicated that AMC ≥ 0.4 × 10^9^/L had a negative impact on progression-free survival (PFS), but not OS [34].

Other investigations have indicated that lymphopenia may influence clinical outcomes. In three separate studies of del(5q), non/del(5q), and low-risk IPSS-R patient with MDS groups, similar results were found, revealing that lymphocytopenia (ALC < 1.2 × 10^9^/L) was independently associated with poorer survival [19,35,36]. The results of our investigation are consistent with those of these reports, emphasizing the negative predictive value of lymphocytopenia in MDS.

Most of the mentioned studies approached the study design using different inclusion criteria and methodology, which could in part explain the discrepancy found in the literature. Unlike some reports that have primarily focused on one subtype of MDS, one risk group, or even included overlapping entities (CMML, MDS/MPN), our patient population encompassed low- and high-risk patients from a single center. The present study on a relatively homogeneous patient population demonstrated a significant impact of monocytopenia and lymphocytopenia on OS, regardless of the IPSS-R risk group. When censored for the usage of disease modifying agents or transplantation, it was demonstrated that both monocytopenia and lymphocytopenia remained a predictor of worse outcome, marking them as an independent risk factor even in higher IPSS-R risk categories, as was showcased in subsequent multivariate analysis. Even more, in patients who received effective treatment, in whom favorable outcomes were generally more frequently observed, monocytopenia was identified as a borderline statistically significant predictor of outcomes. Taking everything into consideration, it is indeed sensible to account for these parameters in future prognostic models.

Certain articles have highlighted the effect of ethnicity on outcomes in MDS [37]. Considering that our study was performed in an ethnically uniform Caucasian cohort, it might prove difficult to apply our findings to other patient populations worldwide. A large international, multiethnic study might provide new data on the effect of monocytopenia and lymphocytopenia in different ethnic populations.

Our study has potential limitations, as only a small percentage of patients received disease-modifying agents or allo-HSCT, due to limited resources and availability at the time of diagnosis. Another shortcoming is the absence of molecular information, therefore hindering the correlation of monocytopenia and lymphocytopenia with molecular risk profile. Additionally, the retrospective nature of this study limited its interpretation in terms of general use and in large cohorts. These findings should be confirmed in prospective studies using real-world clinical trial data.

From a broader perspective, in certain inherited myeloid disorders with GATA2 deficiency [38], monocytopenia has been identified as a hallmark finding. This might provide a basis for further genetic profiling, which could confirm a genetic background of monocytopenia in MDS, leaving room for novel classifications to recognize it as a new subtype.

Despite real-world clinical data accompanied by experimental and clinical trial results, immune and inflammatory pathway-targeting therapies remain underutilized in MDS treatment.

Recent trials on novel targeted drugs have provided mixed results. Drugs such as luspatercept and imetelstat have an important impact on lowering the transfusion burden in lower-risk MDS [39,40]. Moreover, with the growing understanding of inflammasome pathway pathology and their role in promoting self-renewed inflammation that can develop into cellular death and disruption of normal hematopoiesis [41], targeting key signaling mediators such as IRAK1 and IRAK4, as well as ligands and receptors, including S100A9, CD33, IL-1RAP, TGF-β and IL1B, has laid the groundwork for new immune-based treatment of MDS [9]. Monocytes in low-risk MDS groups have been implicated with the increased transcription of pro-inflammatory genes and the associated production of cytokines such as S100A9, IL-1B and NLRP3 further emphasizing their influence on the dysregulation of the normal bone marrow niche. Promising examples of immunotherapy include canakinumab, an IL-1B antibody which has demonstrated effects in reinstating hematopoietic balance in SF3B1 mutated MDS in vitro models [42]. However, the preliminary results of most novel trials have failed to demonstrate outcome benefits, especially in the case of OS, in higher-risk patients. In part, these unsatisfactory findings could be explained by the complexity and redundancy of immune- and inflammation-related pathways and their modular nature of signaling, leading to compensatory buffer effects that counteract the use of single-target agents. Another challenge emerges from unharmonized clinical trial designs that do not effectively translate the novel knowledge of MDS biology into improved outcomes [43,44].

It is safe to assume that better selection of patient subgroups at presentation based on novel immunological data, revised clinical trial designs, and implementation of multi-target combination therapies will create a landscape for breakthrough outcomes [10].

## 5. Conclusions

Our findings suggest that ALC and AMC can serve as readily accessible and verifiable prognostic tools in MDS at presentation. Combined with IPSS-R, these markers may provide additional prognostic insights, enabling better risk stratification in MDS patients who could benefit from future immunotherapies.

## Figures and Tables

**Table 1:** Clinical and laboratory characteristics in 217 patients with myelodysplastic syndrome stratified by the absolute lymphocyte count and absolute monocyte count. (# Mann–Whitney U).

Characteristics	Value; % n = 217	AMC < 0.3 × 10^9^/L (n = 133/61.3; %)	AMC ≥ 0.3 × 10^9^/L (n = 84/38.7; %)	*p* Value	ALC < 1.2 × 10^9^/L (n = 77/35.5; %)	ALC ≥ 1.2 × 10^9^/L (n = 140/64.5; %)	*p* Value
Age (median yrs, range)	70 (28–92)	70 (28–92)	71 (33–89)	0.754 #	74 (31–91)	69 (28–92)	0.003 #
Sex: (M/F)	117/100	69/64	48/36	0.548	44/33	73/67	0.513
Hgb (g/L), median (range)	91 (50–147)	92 (50–147)	88 (52–147)	0.797 #	94 (50–147)	90 (52–147)	0.321 #
Hgb < 100 g/L, n (%)	139 (64)	87	52	0.546	45	94	0.201
WBC ×10^9^/L (median; range)	3.4 (0.8–11.5)	2.7 (0.8–10)	5.0 (2.2–11.5)	0.0001 #	2.8 (0.8–9.2)	3.6 (1.3–11.5)	0.0001 #
WBC < 4 × 10^9^/L, n (%)	141 (65)	110	31	0.0001 #	63	78	0.001 #
Plt (×10^9^/L), median (range)	112 (2–583)	104 (2–583)	125 (4–574)	0.438 #	93 (8–455)	125 (2–583)	0.084 #
Plt < 100 × 10^9^/L, n (%)	100 (46.1)	66	34	0.312	40	60	0.126
Plt < 50 × 10^9^/L, n (%)	50 (23)	30	20	0.472	16	34	0.341
Absolute neutrophil count (×10^9^/L), median (range)	1.4 (0.1–7.68)	1.02 (0.08–7.68)	2.19 (0.26–6.72)	0.0001 #	1.44 (0.08–7.68)	1.44 (0.15–6.72)	0.621 #
Absolute lymphocyte count (×10^9^/L), median (range)	1.4 (0.09–3.65)	1.29 (0.09–3.42)	1.62 (0.25–3.65)	0.0001 #	0.82 (0.09–1.19)	1.65 (1.0–3.65)	/
Absolute monocyte count (×10^9^/L), median (range)	0.22 (0–0.99)	0.13 (0–0.29)	0.55 (0.3–0.99)	/	0.18 (0–0.98)	0.23 (0–0.99)	0.081 #
PB blasts (% median, range)	0 (0–13)	0 (0–13)	0 (0–7)	0.597 #	0 (0–13)	0 (0–9)	0.585 #
BM blasts (% median, range)	6 (1–18)	6 (1–18)	3.5 (1–18)	0.005 #	6 (1–18)	5.5 (1–18)	0.489 #

**Table 2:** Distribution of MDS types and outcomes in 217 patients with myelodysplastic syndrome stratified by the absolute lymphocyte count and absolute monocyte count. (♦ Chi square; # Mann–Whitney U).

	Characteristics	Value; % n = 217	AMC < 0.3 × 10^9^/L (n = 133/61.3; %)	AMC ≥ 0.3 × 10^9^/L (n = 84/38.7; %)	*p* Value	ALC < 1.2 × 10^9^/L (n = 77/35.5; %)	ALC ≥ 1.2 × 10^9^/L (n = 140/64.5; %)	*p* Value
WHO 2016, n (%))	MDS-SLD	28 (12.9)	16 (57.1)	12 (42.9)	0.223 ♦ 0.414 #	13 (46.4)	15 (53.6)	0.069 ♦ 0.805 #
	MDS-MLD	64 (29.5)	38 (59.3)	26 (40.7)		24 (37.5)	40 (62.5)	
	MDS-SLD/MLD-RS	12 (5.5)	4 (33.3)	8 (66.7)		0 (0)	12 (100)	
	MDS(del5q)	8 (3.7)	4 (50)	4 (50)		3 (37.5)	5 (62.5)	
	MDS-EB-1	44 (20.3)	27 (61.4)	17 (38.6)		11 (25)	33 (75)	
	MDS-EB-2	61 (28.1)	44 (72.1)	17 (27.9)		26 (42.6)	35 (57.4)	
	MDS-U	0	0	0		0	0	
IPSS-R, n (%)	Very low	22 (10.1)	11 (50)	11 (50)	0.007 ♦ 0.001 #	12 (54.5)	10 (45.5)	0.336 ♦ 0.515 #
	Low	60 (27.7)	28 (46.7)	32 (53.3)		19 (31.7)	41 (68.3)	
	Intermediate	69 (31.8)	43 (62.3)	26 (37.7)		23 (33.3)	46 (66.7)	
	High	44 (20.3)	35 (79.5)	9 (20.5)		14 (31.8)	30 (68.2)	
	Very high	22 (10.1)	16 (72.7)	6 (27.3)		9 (40.9)	13 (59.1)	
Cytogenetics	Very good	4 (1.8)	2 (50)	2 (50)	0.006 ♦ 0.002 #	4 (100)	0	0.107 ♦ 0.555 #
	Good	172 (79.3)	96 (55.8)	76 (44.2)		58 (33.7)	114 (66.3)	
	Intermediate	24 (11)	22 (91.7)	2 (8.3)		9 (37.5)	15 (62.5)	
	Poor	5 (2.3)	3 (60)	2 (40)		2 (40)	3 (60)	
	Very poor	12 (5.6)	10 (83.3)	2 (16.7)		4 (33.3)	8 (66.7)	
	Transfusion dependent, n (%)	54 (24.9)	39 (72.2)	15 (27.8)	0.049 ♦	22 (40.7)	32 (59.3)	0.382 ♦
	Progression of disease and leukemic transformation (%)	40 (18.4)	28 (70)	12 (30)	0.210 ♦	8 (20)	32 (80)	0.016 ♦
	Deceased (%)	96 (44.2)	71 (73.9)	25 (26.1)	0.0001 ♦	39 (40.6)	57 (59.4)	0.158 ♦

**Table 3:** (**A**) Multivariate analysis (Cox proportional hazard model) with IPSS-R stratified risk (categorical values from very low to very high risk), monocytopenia, lymphocytopenia and DMAs/HSCT in unselected MDS cohort. (**B**) Multivariate analysis (Cox proportional hazard model) with IPSS-R > 3 (very low and low vs. intermediate and high risk) and monocytopenia and lymphocytopenia in selected MDS cohort including patients treated without disease modifying drugs or transplant.

(**A**)				
**n = 217**	**Beta**	***p* Value**	**HR**	**CI 95%**
IPSS-R (risk groups)	0.4795	0.0000	1.6153	1.3396–1.9478
AMC < 0.3 × 10^9^/L	0.5472	0.0237	1.7284	1.0758–2.7768
ALC < 1.2 × 10^9^/L	0.5903	0.0060	1.8045	1.1845–2.7492
DMAs/HSCT	1.3399	0.0000	3.8190	2.1716–6.7160
(**B**)				
**n = 157**	**Beta**	***p* Value**	**HR**	**CI 95%**
IPSS-R > 3	0.5612	0.0000	2.9273	1.7528–4.8886
AMC < 0.3 × 10^9^/L	0.0434	0.0329	1.7065	1.0444–2.7885
ALC < 1.2 × 10^9^/L	0.0945	0.0184	1.7525	1.0993–2.7937

## Data Availability

The data that support the findings of this study are available on request from the corresponding author.

## Abbreviations

The following abbreviations are used in this manuscript:

ALC	Absolute Lymphocyte Count
allo-HSCT	Allogeneic Hematopoietic Stem Cell
AML	Acute Myeloid Leukemia
ANC	Absolute Neutrophil Count
ASCT	Allogeneic Stem Cell Transplantation
BM	Bone Marrow
CMML	Chronic Myelomonocytic Leukemia
ICC	International Consensus Classification
IPSS	International Prognostic Scoring System
IPSS-M	Molecular International Prognostic Scoring System
IPSS-R	Revised International Prognostic Scoring System
KW	Kruskal–Wallis test
MDS	Myelodysplastic Syndrome(s)
MPN	Myeloproliferative Neoplasm
OS	Overall Survival
MWU	Mann–Whitney U test
PB	Peripheral Blood
PFS	Progression-Free Survival
WBC	White Blood Cell Count
WHO	World Health Organization

## References

[1] Khoury J.D., Solary E., Abla O., Akkari Y., Alaggio R., Apperley J.F., Bejar R., Berti E., Busque L., Chan J.K.C. (2022). The 5th edition of the World Health Organization Classification of Haematolymphoid Tumours: Myeloid and Histiocytic/Dendritic Neoplasms. Leukemia.

[2] Arber D.A., Orazi A., Hasserjian R.P., Borowitz M.J., Calvo K.R., Kvasnicka H.-M., Wang S.A., Bagg A., Barbui T., Branford S. (2022). International Consensus Classification of Myeloid Neoplasms and Acute Leukemias: Integrating morphologic, clinical, and genomic data. Blood.

[3] Arber D.A., Orazi A., Hasserjian R., Thiele J., Borowitz M.J., Le Beau M.M., Bloomfield C.D., Cazzola M., Vardiman J.W. (2016). The 2016 revision to the World Health Organization classification of myeloid neoplasms and acute leukemia. Blood.

[4] Fenaux P., Haase D., Santini V., Sanz G., Platzbecker U., Mey U., ESMO Guidelines Committee (2021). Myelodysplastic syndromes: ESMO Clinical Practice Guidelines for diagnosis, treatment and follow-up. Ann. Oncol..

[5] Greenberg P., Cox C., LeBeau M.M., Fenaux P., Morel P., Sanz G., Sanz M., Vallespi T., Hamblin T., Oscier D. (1997). International scoring system for evaluating prognosis in myelodysplastic syndromes. Blood.

[6] Greenberg P.L., Tuechler H., Schanz J., Sanz G., Garcia-Manero G., Solé F., Bennett J.M., Bowen D., Fenaux P., Dreyfus F. (2012). Revised international prognostic scoring system for myelodysplastic syndromes. Blood.

[7] Bernard E., Tuechler H., Greenberg P.L., Hasserjian R.P., Ossa J.E.A., Nannya Y., Devlin S.M., Creignou M., Pinel P., Monnier L. (2022). Molecular International Prognostic Scoring System for Myelodysplastic Syndromes. NEJM Evid..

[8] Winter S., Shoaie S., Kordasti S., Platzbecker U. (2020). Integrating the “Immunome” in the Stratification of Myelodysplastic Syndromes and Future Clinical Trial Design. J. Clin. Oncol..

[9] Trowbridge J.J., Starczynowski D.T. (2021). Innate immune pathways and inflammation in hematopoietic aging, clonal hematopoiesis, and MDS. J. Exp. Med..

[10] Simoni Y., Chapuis N. (2022). Diagnosis of Myelodysplastic Syndromes: From Immunological Observations to Clinical Applications. Diagnostics.

[11] Kouroukli O., Symeonidis A., Foukas P., Maragkou M.-K., Kourea E.P. (2022). Bone Marrow Immune Microenvironment in Myelodysplastic Syndromes. Cancers.

[12] Tentori C.A., Gregorio C., Robin M., Gagelmann N., Gurnari C., Ball S., Berrocal J.C.C., Lanino L., D’AMico S., Spreafico M. (2024). Clinical and Genomic-Based Decision Support System to Define the Optimal Timing of Allogeneic Hematopoietic Stem-Cell Transplantation in Patients with Myelodysplastic Syndromes. J. Clin. Oncol..

[13] Sauta E., Robin M., Bersanelli M., Travaglino E., Meggendorfer M., Zhao L.-P., Berrocal J.C.C., Sala C., Maggioni G., Bernardi M. (2023). Real-World Validation of Molecular International Prognostic Scoring System for Myelodysplastic Syndromes. J. Clin. Oncol..

[14] Ray-Coquard I., Cropet C., Van Glabbeke M., Sebban C., Le Cesne A., Judson I., Tredan O., Verweij J., Biron P., Labidi I. (2009). Lymphopenia as a prognostic factor for overall survival in advanced carcinomas, sarcomas, and lymphomas. Cancer Res..

[15] Tadmor T. (2013). Does monocyte count have prognostic significance in cancer?. Leuk. Res..

[16] Jakovic L.R., Mihaljevic B.S., Andjelic B.M., Bogdanovic A.D., Perunicic Jovanovic M.D., Babic D.D., Bumbasirevic V.Z. (2016). Prognostic value of lymphocyte/monocyte ratio in advanced Hodgkin lymphoma: Correlation with International Prognostic Score and tumor associated macrophages. Leuk. Lymphoma.

[17] Sáenz J., Izura J., Manrique A., Sala F., Gaminde I. (2001). Early prognosis in severe sepsis via analyzing the monocyte immunophenotype. Intensive Care Med..

[18] Elliott M.A., Verstovsek S., Dingli D., Schwager S.M., Mesa R.A., Li C.Y., Tefferi A. (2007). Monocytosis is an adverse prognostic factor for survival in younger patients with primary myelofibrosis. Leuk. Res..

[19] Silzle T., Blum S., Schuler E., Kaivers J., Rudelius M., Hildebrandt B., Gattermann N., Haas R., Germing U. (2019). Lymphopenia at diagnosis is highly prevalent in myelodysplastic syndromes and has an independent negative prognostic value in IPSS-R-low-risk patients. Blood Cancer J..

[20] Saeed L., Patnaik M.M., Begna K.H., Al-Kali A., Litzow M.R., Hanson C.A., Ketterling R.P., Porrata L.F., Pardanani A., Gangat N. (2017). Prognostic relevance of lymphocytopenia, monocytopenia and lymphocyte-to-monocyte ratio in primary myelodysplastic syndromes: A single center experience in 889 patients. Blood Cancer J..

[21] Silzle T., Blum S., Kasprzak A., Nachtkamp K., Rudelius M., Hildebrandt B., Götze K.S., Gattermann N., Lauseker M., Germing U. (2023). The Absolute Monocyte Count at Diagnosis Affects Prognosis in Myelodysplastic Syndromes Independently of the IPSS-R Risk Score. Cancers.

[22] Diamantopoulos P.T., Charakopoulos E., Symeonidis A., Kotsianidis I., Viniou N.-A., Pappa V., Pontikoglou C., Tsokanas D., Drakos G., Kourakli A. (2022). Real world data on the prognostic significance of monocytopenia in myelodysplastic syndrome. Sci. Rep..

[23] Nordic MDS Group (2021). Guidelines for the Diagnosis and Treatment of Myelodysplastic Syndrome and Chronic Myelomonocytic Leukemia [Internet]. https://www.nmds.org/attachments/article/121/NMDSG_guidelines_dec2021.pdf.

[24] Clinical and Laboratory Standards Institute (2007). Reference Leukocyte Differential Count (Proportional) and Evaluation of Instrument Methods.

[25] Lee S., Erber W.N., Porwit A., Tomonaga M., Peterson L.C., International Council for Standardization In Hematology (2008). ICSH guidelines for the standardization of bone marrow specimens and reports. Int. J. Lab. Hematol..

[26] Itzykson R., Fenaux P., Bowen D., Cross N.C., Cortes J., De Witte T., Germing U., Onida F., Padron E., Platzbecker U. (2018). Diagnosis and Treatment of Chronic Myelomonocytic Leukemias in Adults: Recommendations From the European Hematology Association and the European LeukemiaNet. Hemasphere.

[27] Bain B.J. (1996). Ethnic and sex differences in the total and differential white cell count and platelet count. J. Clin. Pathol..

[28] Merz A.M.A., Platzbecker U. (2025). Treatment of lower-risk myelodysplastic syndromes. Haematologica.

[29] Velegraki M., Papakonstanti E., Mavroudi I., Psyllaki M., Tsatsanis C., Oulas A., Iliopoulos I., Katonis P., Papadaki H.A. (2013). Impaired clearance of apoptotic cells leads to HMGB1 release in the bone marrow of patients with myelodysplastic syndromes and induces TLR4-mediated cytokine production. Haematologica.

[30] Pang W.W., Pluvinage J.V., Price E.A., Sridhar K., Arber D.A., Greenberg P.L., Schrier S.L., Park C.Y., Weissman I.L. (2013). Hematopoietic stem cell and progenitor cell mechanisms in myelodysplastic syndromes. Proc. Natl. Acad. Sci. USA.

[31] Bahmani F., Shayanmanesh M., Safari M., Alaei A., Pouriafar Y., Rasti Z., Zaker F., Rostami S., Damerchiloo F., Safa M. (2025). Bone marrow microenvironment in myelodysplastic neoplasms: Insights into pathogenesis, biomarkers, and therapeutic targets. Cancer Cell Int..

[32] Kordasti S.Y., Afzali B., Lim Z., Ingram W., Hayden J., Barber L., Matthews K., Chelliah R., Guinn B., Lombardi G. (2009). IL-17-producing CD4(+) T cells, pro-inflammatory cytokines and apoptosis are increased in low risk myelodysplastic syndrome. Br. J. Haematol..

[33] Kasprzak A., Assadi C., Nachtkamp K., Rudelius M., Haas R., Giagounidis A., Götze K., Gattermann N., Germing U. (2023). Monocytosis at the time of diagnosis has a negative prognostic impact in myelodysplastic syndromes with less than 5% bone marrow blasts. Ann. Hematol..

[34] Qu H., Chu J., Wang L., Zhang J., Han J., Li Z., Hou H., Wang Y., Liu Y., Wu H. (2024). Platelet-to-lymphocyte ratio and absolute monocyte count have prognostic potential in primary myelodysplastic neoplasms. Int. J. Lab. Hematol..

[35] Holtan S.G., Santana-Davila R., DeWald G.W., Khetterling R.P., Knudson R.A., Hoyer J.D., Chen D., Hanson C.A., Porrata L., Tefferi A. (2008). Myelodysplastic syndromes associated with interstitial deletion of chromosome 5q: Clinicopathologic correlations and new insights from the pre-lenalidomide era. Am. J. Hematol..

[36] Jacobs N.L., Holtan S.G., Porrata L.F., Markovic S.N., Tefferi A., Steensma D.P. (2010). Host immunity affects survival in myelodysplastic syndromes: Independent prognostic value of the absolute lymphocyte count. Am. J. Hematol..

[37] Goksu S.Y., Ozer M., Goksu B.B., Wang R., Khatib J., Patel P.A., Vusirikala M., Cole S., Seyhanli A., Collins R.H. (2022). The impact of race and ethnicity on outcomes of patients with myelodysplastic syndromes: A population-based analysis. Leuk. Lymphoma.

[38] Mir M.A., Kochuparambil S.T., Abraham R.S., Rodriguez V., Howard M., Hsu A.P., Jackson A.E., Holland S.M., Patnaik M.M. (2015). Spectrum of myeloid neoplasms and immune deficiency associated with germline GATA2 mutations. Cancer Med..

[39] Platzbecker U., Della Porta M.G., Santini V., Zeidan A.M., Komrokji R.S., Shortt J., Valcarcel D., Jonasova A., Dimicoli-Salazar S., Tiong I.S. (2023). Efficacy and safety of luspatercept versus epoetin alfa in erythropoiesis-stimulating agent-naive, transfusion-dependent, lower-risk myelodysplastic syndromes (COMMANDS): Interim analysis of a phase 3, open-label, randomised controlled trial. Lancet.

[40] Platzbecker U., Santini V., Fenaux P., Sekeres M.A., Savona M.R., Madanat Y.F., Díez-Campelo M., Valcárcel D., Illmer T., Jonášová A. (2024). Imetelstat in patients with lower-risk myelodysplastic syndromes who have relapsed or are refractory to erythropoiesis-stimulating agents (IMerge): A multinational, randomised, double-blind, placebo-controlled, phase 3 trial. Lancet.

[41] Sallman D.A., List A. (2019). The central role of inflammatory signaling in the pathogenesis of myelodysplastic syndromes. Blood.

[42] Schneider M., Rolfs C., Trumpp M., Winter S., Fischer L., Richter M., Menger V., Nenoff K., Grieb N., Metzeler K.H. (2023). Activation of distinct inflammatory pathways in subgroups of LR-MDS. Leukemia.

[43] Mina A., McGraw K.L., Cunningham L., Kim N., Jen E.Y., Calvo K.R., Ehrlich L.A., Aplan P.D., Garcia-Manero G., Foran J.M. (2025). Advancing drug development in myelodysplastic syndromes. Blood Adv..

[44] Sekeres M.A., Kim N., DeZern A.E., Norsworthy K.J., Garcia J.S., de Claro R.A., Theoret M.R., Jen E.Y., Ehrlich L.A., Zeidan A.M. (2023). Considerations for Drug Development in Myelodysplastic Syndromes. Clin Cancer Res..

